# Design considerations for a wearable monitor to measure finger posture

**DOI:** 10.1186/1743-0003-2-5

**Published:** 2005-03-01

**Authors:** Lisa K Simone, Derek G Kamper

**Affiliations:** 1 Kessler Medical Rehabilitation Research and Education Corporation, West Orange, NJ, USA; 2 Sensory Motor Performance Program, Rehabilitation Institute of Chicago, Chicago, IL, USA

**Keywords:** Finger flexion, Range of Motion, sensors, home monitoring

## Abstract

**Background:**

Objective measures of hand function as individuals participate in home and community activities are needed in order to better plan and evaluate rehabilitation treatments. Traditional measures collected in the clinical setting are often not reflective of actual functional performance. Recent advances in technology, however, enable the development of a lightweight, comfortable data collection monitor to measure hand kinematics.

**Methods:**

This paper presents the design analysis of a wearable sensor glove with a specific focus on the sensors selected to measure bend. The most important requirement for the glove is easy donning and removal for individuals with significantly reduced range of motion in the hands and fingers. Additional requirements include comfort and durability, cost effectiveness, and measurement repeatability. These requirements eliminate existing measurement gloves from consideration. Glove construction is introduced, and the sensor selection and glove evaluation process are presented.

**Results:**

Evaluation of commercial bend sensors shows that although most are not appropriate for repeatable measurements of finger flexion, one has been successfully identified. A case study for sensor glove repeatability using the final glove configuration and sensors does show a high degree of repeatability in both the gripped and flat hand positions (average coefficient of variability = 2.96% and 0.10%, respectively).

**Conclusion:**

Measuring functional outcomes in a portable manner can provide a wealth of information important to clinicians for the evaluation and treatment of movement disorders in the hand and fingers. This device is an important step in that direction as both a research and an evaluation method.

## Background

Rehabilitation research has recently begun to emphasize the use of objective outcome measures to assess the efficacy of rehabilitation treatment or intervention [1]. These goals could be greatly furthered through the development of wearable measurement systems that provide an opportunity to evaluate how the individual participates in home and community activities. Information collected in this manner can provide a more realistic snapshot of activity and function than traditional methods which restrict measurements to the clinical or research site. Data describing actual usage in the home is especially important for the hand as hand movement is so closely tied to performance of functional tasks. In order to understand how individuals truly interact with their environments, we wish to obtain quantitative measures of finger flexion and extension over longer periods of time than traditionally investigated (such as monitoring over a full circadian cycle).

Unfortunately, rehabilitation researchers have very few methods available to objectively evaluate hand use and function outside the clinic, especially for individuals with moderate to severe reduction in range of motion in the hand and fingers. Joint range of motion (ROM) is a primary measure in hand rehabilitation, and is traditionally assessed using manual goniometry. Manual methods, however, are limited to static measurements. In addition, they can be adversely affected by common issues such as inter- and intra-operator error and operator experience level [2].

In order to objectively measure hand use outside the clinic, the selected method must be both portable and capable of recording continuous streams of data over time. Automated measurement methods can meet these requirements as well as eliminate most operator-related issues. For example, 24-hour monitoring has proven useful for tracking parameters such as heart rate and blood pressure, and the same concept can be extended to other useful parameters, although currently no wearable devices are available to measure finger posture in a similar manner. Practical medical applications can include providing input for virtual reality therapy, measuring hand function in the planning of rehabilitation therapies, or evaluating the outcome of interventions under more realistic conditions.

Sensor gloves have been proposed to provide automatic measurements of finger and joint position during different activities, with the virtual reality industry continuing to drive the market for sensor gloves [3]. For example, Fifth Dimension Technologies (5DT, Irvine, CA) produces a 5-sensor and a 16-sensor version wireless sensor glove which transmits data to a nearby computer. Two joints per finger are captured with these expensive devices (proximal interphalangeal joint (PIP) and metacarpophalangeal joint (MCP)) using fiber optic sensors. The White Hand Group (Mississippi State University) is developing a lower-cost (<$500) flexion data glove using fiber optics that has two sensors per finger. The glove is tethered to a computer and is aimed at VR and gaming applications rather than accurate measurement applications.

CyberGlove (Immersion Corporation, San Jose, CA) contains 5 to 22 "patented bend-sensing technology" strain gauges to measure individual joint movements. This glove, however, is very expensive and difficult for stroke survivors to don. The Essential Reality (Mineola, NY) P5 glove also uses bend sensors, Abrams/Gentile flex sensors (wired flexion measurements), and infrared (IR) emitters (line-of-site wireless position and rotation measurements). The flex sensors are attached to each finger by rings between the proximal and distal interphalangeal joints (DIP). While price of this glove is appealing (<$200), the glove is not portable and requires the wearer to keep the top of the glove always facing a fixed antenna IR receiver.

The Humanglove™ (Humanware S.R.L., Pisa, Italy) is a flexible glove with 20 Hall effect sensors to measure bend. The Humanglove was evaluated for feasibility and repeatability in finger range of motion in all joints; work continues to establish the measurement accuracy [2].

Several research gloves have been reported with no clinical results. Karlsson et al. [4] determined finger flexion by measuring the pressure changes in airtight polyvinyl tubes on three fingers. Zurbrügg [5] measured flexion using potentiometers mounted on the back of the hand, although the construction is not durable for long term measurements. Hofmann and Henz [6] used inductive length encoders attached to a cotton glove to measure flexion and gestures in real time. The glove is not easy to put on, and the sensors can move around relative to the joint position. Jurgens et al. [7] proposed an innovative method using electrically conducting ink printed on a flexible polyester plastic for a low cost solution, although disadvantages include extreme sensitivity to small changes in temperature, and a moderately slow response time.

The existing glove systems do not meet the needs set forth in our device requirements. Although some gloves are lightweight, others such as force feedback and exoskeleton based gloves are too heavy and bulky for home use by individuals with hand impairments. Most are too expensive or require custom sizing to reduce errors; measurement errors are decreased when gloves fit more snugly [2]. However, donning a tight glove can be difficult to impossible for some individuals with movement disorders in the hand. Full gloves have other limitations as well. Actions such as wrist flexion and rotation in full fabric gloves can cause the glove material to move over the skin. In this case, friction can prevent the material from returning completely to the original position, leaving the sensors located in different positions over the joints [2]. This source of error is manifested as a drift in measured bend that is difficult to detect, characterize, and eliminate from recorded data streams.

The environment proposed for the glove use is also very challenging. A majority of the existing methods are not appropriate for wearable measurements in the home and community, and none are designed for use with clinical and rehabilitation populations who may exhibit significant range of motion restriction in the fingers.

The long term goal of this project, therefore, is to design, build, and clinically evaluate a novel low-cost wireless device to measure hand and finger activity while individuals participate in normal home and community activities. It will be used to study the loss of hand function that can occur following neurological disorders such as stroke and traumatic brain injury, and to evaluate how treatments can improve an individual's ability to function in the home and community environments.

This article describes the design process for the development of the glove, discussing wearability issues such as comfort, durability and weight and focusing on sensor characterization and selection. The results of an initial evaluation of measurement repeatability while wearing the glove are presented. Appropriate characterization of the glove must occur in two phases: evaluation of the sensors separately, and then evaluation of the entire glove after appropriate sensors have been identified and characterized. Full repeatability results and measurement accuracy will be reported in the future.

## Methods

The creation of effective custom measurement systems requires detailed attention to the requirements and design analysis phases of the development process. While it may be tempting to solve multiple problems with one system, this often leads to overly complicated devices that take too long to complete, and may not actually meet the core requirements. To avoid this scenario, the sensor glove project focused specifically on a set of core requirements presented below, and pursued a multi-step analysis of the design to ensure the requirements were being appropriately addressed. The steps include an analysis of glove construction methods, characterisation of sensors, and exploration of sensor repeatability and accuracy on the bench and during subject trials.

The primary requirements fall into four categories.

1) Donning and Removing: The glove must be easy to don and remove for individuals having reduced range of motion in the hand and fingers.

2) Comfort and Durability: The glove must be lightweight and unobtrusive, and permit comfortable wearing for at least 24 hours. It must not restrict range of motion or represent a snag hazard during use.

3) Function: The glove must detect a wide range of activities, including fine motor activities such as writing. Measurements must be accurate and repeatable. The sensors must not move around, but remain in the same position with respect to the joints and phalanges over time. The system must allow the performance of normal daily activities, although use in water is not required. Measurements must be repeatable with an error no more than 5% of full scale.

4) Cost: Manufacturing cost is an important consideration for several reasons. An inexpensive device allows several to be deployed simultaneously to perform research studies more quickly. A low-cost glove can be considered disposable for sanitary reasons. Finally, wearers may unknowingly limit or modify their hand motions and activities in an attempt to protect an expensive device. A low-cost alternative ensures that more realistic and representative data are captured and recorded.

As noted, a variety of sensors have been employed to measure joint angle, including strain gauges or bend sensors, fiber optics, pneumatics, or Hall effect sensors. While each has advantages and disadvantages, the requirements for our glove preclude the use of all but the bend sensors. Using fiber optics to measure bend requires a light source such as a light emitting diode and a photo detector. The amount of bend is proportional to the attenuation of detected light in specially treated sections of fiber that pass over the tops of the finger joints. Disadvantages of this method include the complexity of glove construction and price. Hall effect sensors, which detect magnetic fields, and can be configured as proximity sensors to provide a linear output proportional to distance from a magnetic source. By placing a series of sensors on the back of a glove in a predefined pattern, the joint angle can be computed from the changing field strengths measured by the sensors when the fingers bend. While these devices are small, the resulting glove can be somewhat bulky and will require a magnetic source, adding to overall weight. Interference from other electromagnetic sources is also a concern. Strain gauges detect stretch produced by joint rotation. They may have very high accuracy, but are expensive and often delicate.

Bend sensors offer a lightweight and inexpensive alternative. These sensors are thin flexible membranes that change resistance when bent; increasing bend angle is generally associated with increased measured resistance. One or more layers of carbon and a conductive material are applied over a thin substrate. Depending on the sensor type, bending motion forces conductive particles further apart, increasing the resistance to current flow. These sensors are popular for detecting obstacles and measuring large changes in bend angle, and are proposed for accurate measurement of finger posture. However, most exhibit a time-varying creep behavior when held in a fixed bent position that reduces the accuracy of measurement. We sought to find bend sensors that would be feasible.

### Glove Construction Methods

In order to explore the first two requirements, ease of donning and comfort/durability, several prototype glove systems were created in order to identify the best materials and construction methods to satisfy these requirements. Several materials were evaluated, including blends of Lycra^®^, Nylon and cotton. The material must exhibit stretch so that finger motion and bending are not restricted. While some blends resisted finger motions, most were flexible enough that the wearer could forget the glove was on. A discussion of the process and final selection of glove materials and application method can be found elsewhere [8].

The final glove material is a 93% Lycra^® ^/ 7% Nylon blend; it is used to create thin sleeves into which the sensors are inserted. One sleeve is attached to the back of each finger in order to locate the sensor directly over each joint; the optimal adhesive is very thin, double-sided toupee tape. Applying the sensors to the back of the fingers, rather than using a traditional glove that must be donned, allows easy application and removal for individuals who cannot fully open all fingers to put on a traditional glove. Total glove cost without sensors is less than $2.50.

Comfort and durability were evaluated over a 24 hour period. The glove survived intact and did not impede any activities other than showering and tucking in a shirt. Because the sensors are attached to the back of the hand, the palmar surface of the hand is uncovered and free of obstruction, leaving the sense of touch intact. While only one individual was used to narrow down the different prototype ideas, 24 individuals (12 with brain injury and 12 healthy controls) are currently participating in a study to more fully evaluate the glove configuration and performance.

### Sensor Repeatability

An early decision was made to use an inexpensive bend sensor as the sensing element for its low profile, light weight, and low cost. While the first prototype glove has only 5 sensors, the design provides the flexibility to add additional sensors for all joints and for finger adduction/abduction. Sensors from several manufacturers were characterized in order to determine if measurements were repeatable and if large changes in finger posture and fine motor control could be captured. In addition, the calibration relationship between bend angle and measured resistance was evaluated.

The importance of repeatability testing cannot be overemphasized. The sensors were evaluated separately before being incorporated into the sensor glove. Two types of tests were performed using a set of tubes of known diameter: 1) determination of full scale and the resistance-bend relationship (using tube diameters: 4", 3", 2", and 1.5", 1" and 0.75"), and 2) evaluation of measurement repeatability (using the 3" diameter tube). A physical guide was placed on each tube to ensure that the sensor was placed in the same location on the tube each time it was applied. From four to ten sensors from each manufacturer were evaluated. To test the sensors, each was initially placed flat on the table for several seconds, then bent over a single calibration tube for 30 to 60 seconds, and then placed flat again. Resistance readings were taken during each phase. The expected profile for this activity should look like a rectangular wave between two resistance values: a lower resistance value in the flat position, then the higher value when bent on the tube, and ending with the original lower flat value. Multiple measurements were taken to evaluate the variation in sensor outputs, and to construct a bend resistance versus tube diameter relationship that could be used for calibration purposes.

Full scale is determined from the endpoints of the calibration relationship. The minimum measured resistance value corresponds to the flat position, and the maximum resistance value is represented by the resistance measured on the 0.75" calibration tube. The maximum value is an estimate of what would be observed in human subjects because the bending radius over a finger joint is not exactly replicated by bending over different diameter tubes; however, making this assumption allows the different sensors to be compared to one another for selection purposes. Full scale error is computed as the percent change in resistance measured while a sensor is fixed unmoving on a calibration tube with respect to the full scale range.

A second error is also reported, and is calculated as the percent change in the peak sensor resistance with respect to the magnitude of the step function rise in resistance when the sensor is positioned on a calibration tube. This error calculation was also selected for comparison because a number of everyday activities such as grasping and moving objects find the hand in roughly this position.

As discussed in the results section, additional analysis was performed to explore an unexpected time-varying behavior of the sensors. Collection of these data and of the data described in the next section was performed using a host computer with an 8 channel 16-bit A/D card. The sensors were connected to an interface box and data on all sensors was collected at 100 Hz using LabView (National Instruments, Austin, TX). Analysis was performed using Microsoft Excel.

### Sensor Glove Repeatability

A major concern in developing a measurement method is that measurements are repeatable. If the bend sensors and the configuration of these sensors on the fingers will be used for several measurements during the same session, or for measurements over several sessions, then repeatability must be established before the data can be given credence. Rigorous validation of repeatability, however, is often lacking from descriptions of various "data gloves."

One method to evaluate repeatability of sensor glove-type devices has been proposed by Wise [9] and expanded by Dipietro [2]. The method was specifically developed for devices that perform semi- or fully-automated goniometric measurements. Repeatability was established using a custom mold created by each subject. The mold was made while the subject simulated a grip position around a wet mixture of Plaster of Paris. After the mold dried, it was used to ensure that the subject's finger positions were identical for several consecutive gripping actions on the mold. Testing with 5 healthy control subjects revealed errors that led to recommendations for improvements in measurement methodology. Dipietro [2] repeated these procedures with some clarifications in hand position in the evaluation of the Humanglove, and echoed the need for standardized testing protocols for sensor glove devices.

This standardized repeatability testing protocol procedure includes four tests. We will use two, Tests A and C, which focus on repeatability of multiple measurements over a single data collection session. Tests A and C are performed here because the first focus of the glove is for single data collection sessions (which can last up to 24 hours). The protocol used here follows the modifications proposed in [2]; an overview of the tests appears below.

Test A: A roughly cylindrical plaster mold is custom created for each subject to ensure that the fingers are flexed to the same position for each test. The participant clenches the mold for 6 seconds and then releases the mold for 6 seconds. This clench/release cycle is repeated 10 times. Repeatability measurements are taken from each sensor during the clench phases.

Test C: The participant places the hand on a table top and alternately raises the hand and lightly flexes the fingers, and returns the hand to the table top for 6 second each. Repeatability of the flat hand position is explored in this test. In order to achieve repeatability in hand and finger position, and outline of the hand profile is drawn on paper and placed on the table. This cycle is also repeated 10 times.

For each test above, the participant rested for at least 1 minute, and then repeated the entire test. This was done 10 times for both Test A and Test C, for a total of 100 grip/release cycles for each test. Descriptive statistics are computed (mean, standard deviation, and coefficient of variation). The percent coefficient of variation (standard deviation divided by the mean*100%) is used to compare the measurement variability among the five digits and between the two repeatability tests.

## Results and Discussion

### Glove Construction

**Figure 1:**
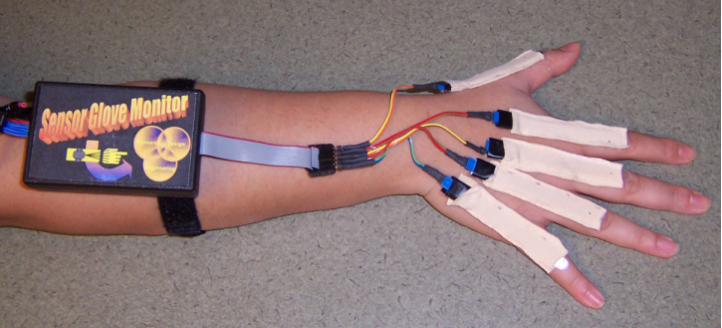
**Prototype of the sensor glove monitor. **The monitor is shown with five sensors placed on the MCP joints. Signal conditioning is contained in the box. The next prototype will include a wireless link for data download to an external computer, enabling the removal of the cable extending out of the back of the box.

Figure 1 shows a prototype of the sensor glove monitor. For the test shown here, one sensor was used to measure flexion of each metacarpophalangeal (MCP) joint. The sensors are located inside the beige sensors sleeves, which are attached to the back of the metacarpals and proximal phalanges. The sensors do not move relative to the joint under measurement. The forearm-mounted box contains signal conditioning. In the next prototype, the box will also contain a wireless transmitter and the cable from the left of the box will be removed, allowing the participant to move around freely. Instead of Velcro bands, a comfortable band of flexible material will hold the box to the forearm. Data will initially be transmitted wirelessly to a nearby laptop computer, and eventually transmitted wirelessly to a waist mounted data recorder. The sensors and glove sleeves weigh less than 7.1 grams; adding the signal conditioning box increases the weight to 85 grams. The final device will have the added weight of a battery and small wireless transmitter.

### Sensor Repeatability

Performing sensor evaluation and characterization early in the design process allowed us to identify several shortcomings of commercial bend sensors and eventually select an appropriate sensor.

**Figure 2:**
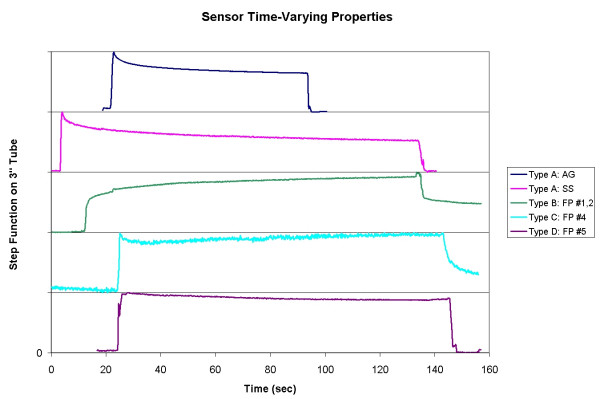
**Sensor time-varying properties. **Each of several sensors is placed on a calibration tube for at least 30 seconds, and then stretched flat on a table in order to verify that a constant relationship exists between bend angle and measured resistance. Ideally, these curves should be flat but a significant time-varying decay renders most unusable for this application. This figure shows several representative curves for the three manufacturers evaluated. AG: Abrams-Gentile sensors, SS: SpectraSymbol sensors, FP: Flexpoint sensors (several types).

The first sensor evaluated was the Abrams-Gentile Entertainment, Inc. (New York, NY) sensor patent #5,086,785. Attempts to measure repeatable bend resistance versus calibration tube diameter failed because the measured resistance decayed over time. The Abrams-Gentile sensor exhibits the most common behavior that we will refer to as Type A behavior, and it appears in Figure 2 as line "AG". The sensor reached a peak resistance value just as it was wrapped around the calibration tube, with an immediate decay in resistance over time. We expected that the sensor values would be constant; however, the drift in measured resistance prevented an accurate and repeatable measurement of bend. To eliminate other potential sources of error, the analysis was repeated on ten other sensors, and the problem finally isolated to the sensors by testing each directly using an ohmmeter.

The average decay in resistance while on the tube was computed. After 30 seconds, the average error for the Abrams-Gentile sensors was 9.5% of full scale, and 24.4% of step function rise resistance (Table 1). The Abrams-Gentile sensor never settled on a final resistance value, but over an extended two-day data collection session continued to slowly decay. While these sensors are appropriate for many applications such as position detectors and indicators of gross movement, we determined that they are not appropriate for accurate and repeatable measurements of finger flexion.

**Table 1:** Sensor decay over time for three sensor types fixed over a 3-inch tube

**% Full Scale**			**% Step Function Rise**			
Time (sec)	Abrams-Gentile	Flexpoint	Time (sec)	Abrams-Gentile	Spectra Symbol	Flexpoint

0	0.0%	0.0%	0	0.0%	0.0%	0.0%
2	3.9%	0.3%	2	9.9%	15.2%	2.6%
4	5.2%	0.3%	4	13.4%	17.8%	3.2%
15	7.9%	0.6%	15	20.4%	25.7%	6.1%
30	9.5%	0.8%	30	24.4%	31.8%	8.9%

The same testing was repeated using sensors from Spectra Symbol (Salt Lake City, UT). Similarly, the step function rise in resistance measured on application to the 3" calibration ring dropped 31.8 % in the first 30 seconds (Figure 2, Type A: SS). Again, the sensor is better suited to sensing a change in angle, rather than the magnitude of the change. A calibration relationship was not explored because the magnitude of the error was so large.

**Figure 3:**
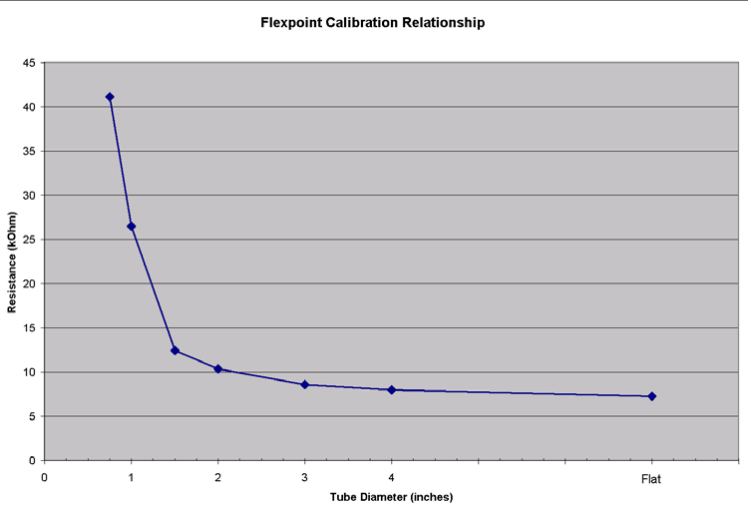
**Resistance – bend relationship of the Flexpoint sensor. **The Flexpoint sensor has a nonlinear relationship between measured resistance and bend diameter, as found by measuring resistance for sensors wrapped around calibration tubes of different diameters. For illustration purposes, the relationship presented here is an average of several sensors; a separate relationship will be measured for each sensor used in the sensor glove.

Six different sensor configurations were evaluated from Flexpoint (South Draper, UT). These included flex sensors with an overlaminate adhered by a pressure sensitive adhesive (sensor #1), with a robust polyimide overlaminate (sensor #2), with no overlamination but with a stiff backer (sensor #3), and an overmolded sensor (sensor #4) for harsh environmental conditions. Representative contours for the 3" calibration test are shown in Figure 2, labelled "FP: #1, 2, 4, and 5" Sensor 3 exhibited the same large decays observed with the Type A Abrams-Gentile and SpectraSymbol sensors shown in the figure. In contrast, sensors 1 and 2 (Type B) responded to the initial fast bend over the tube with a slow rise in resistance that never reached a peak. When the sensors were removed from the tube and placed flat again, the resistance decayed but did not reach baseline values for many minutes. Sensor 4 (Type C) exhibited a fast response to a peak value, dropped 15% of the rise amount and then slowly recovered the 15% over several minutes, but never returned to the baseline value when placed flat at the end of the test. None of these sensors (1–4) is appropriate for the sensor glove. However, in consultation with the company describing our exact needs, a solution was identified. The bend sensors are generally not supplied without some type of protection layer. However, these layers tended to cause the observed decay problems, making these sensors inappropriate for this application. Evaluation of bare sensors (Figure 2, Type D) revealed the initial peak resistance followed by decay; however, the magnitude of the decay after 30 seconds was only 0.8% full scale or 8.9% of step function resistance rise, which is acceptable for this application. The bare version of the sensor is approximately $7.10 in low quantities. The average relationship between bend angle and resistance for 5 sensor trials is shown in Figure 3. The error results from all sensors are shown in Table 1.

Bend sensors are used in a number of university and home projects, despite our findings that most are not repeatable for moderate to fine resolution measurements. Instead, most are appropriate for binary ON/OFF applications, or applications that do not require high resolution or highly repeatable results. Examples include using the sensor as a "whisker" to sense the proximity of an object for collision detection, for detecting large changes in bend angle, or for more unique applications such as adding effects to music [10]. Others report early results for such implementations as measurement of foot flexion for biofeedback [11] although follow-up work on calibration and analysis methods is still pending. We located no references validating and using these sensors, and only one reference that indicated that the Abrams-Gentile sensor was "difficult to work with" [12]. For the measurement device described here, repeatability of measurement is an important criterion. Only the bare Flexpoint sensor was found to be appropriate for measuring fine changes in bend angle in a repeatable manner. If another sensor is used in this application, there is no way to determine the actual bend radius because a wide range of measured resistance values (caused by the decay) correspond to a single bend radius.

### Sensor Glove Repeatability

**Figure 4:**
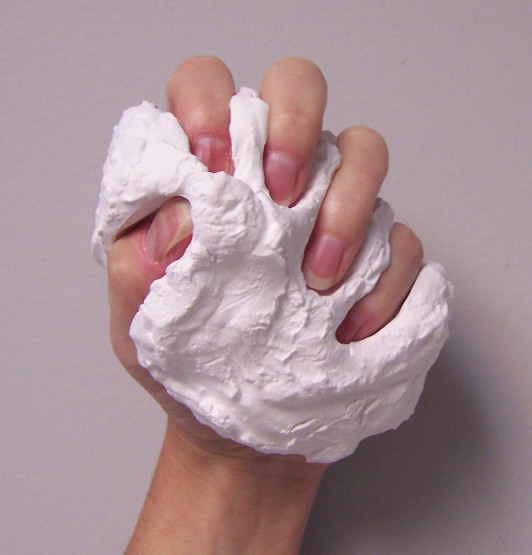
**Grip mold. **The grip mold is custom made for each subject. It provides a repeatable position for the fingers to assume for multiple grip-release activities in order to evaluate repeatability of measurement.

Repeatability testing began with the evaluation of the sensors and final sensor selection, and continues by considering the performance of the entire glove. The sensor glove repeatability testing has been performed with one participant, who is the first in a study that will include repeatability testing for 6 healthy controls. All participants complete an Institutional Review Board consent form and the required HIPAA authorization forms. The plaster mold was created as shown in Figure 4.

**Figure 5:**
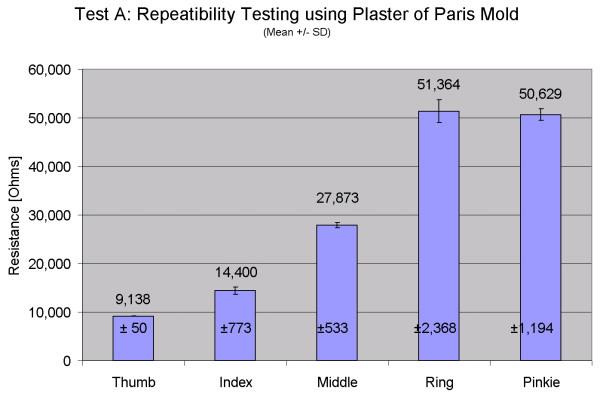
**Repeatability testing of grip position. **Repeatability testing of one participant for the grip test (Test A). Means and standard deviations are shown. Mean values differ because each finger is in a different position when gripping the mold. Repeatability information is contained in the variation around the mean.

The results of Test A reveal the repeatability of measurement in the grip hand position. Coefficient of variability for all five digits is less than 6% (thumb: 0.55%, index: 5.37%, middle: 1.91%, ring: 4.61%, pinkie: 2.36%). Figure 5 shows the mean and standard deviation of measured MCP joint position while gripping the mold 100 times. The mean values indicate the average resistance of the sensors when the fingers are gripping the mold. In this test, the actual mean value of bend is not critical, it just represents the joint position when the mold was made. In addition, these values are not calibrated. For this individual, the thumb MCP is the least bent (having the lowest resistance values) while the ring and pinkie fingers are significantly bent. The repeatability information is located within the very low coefficient of variation, or variation of the measured values about the mean.

**Figure 6:**
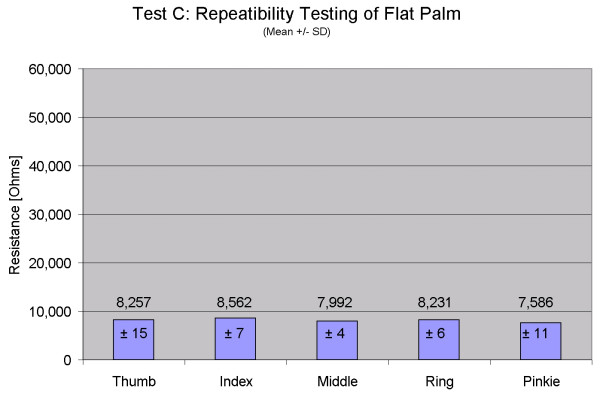
**Repeatability testing of flat hand position. **Repeatability testing of one participant for the flat hand test (Test C). Means and standard deviations are shown. Mean values are similar because all fingers are straight when data collection occurs.

The results for Test C show the repeatability in the flat hand position with the sensors fully extended. Coefficient of variability for all five digits is less than 1% (thumb: 0.18%, index: 0.08%, middle: 0.05%, ring: 0.07%, pinkie: 0.15%). Figure 6 shows the mean and standard deviation of measured MCP joint position while placing the hand flat 100 times. Descriptive statistics and coefficient  of variability for Tests A and C are shown in Table 2. The variation in measurements for the flat hand position is extremely small over the 100 cycles, which is very encouraging considering that the flat hand position is only guided by an outline of the hand on the tabletop. Five additional participants will complete this repeatability testing and results presented in the future.

These results are similar to [2] in that the measurement repeatability was better in the flat hand (Test C) case than in the grip mold case (Test A). Dipietro [2] speculated that the "hand is positioned more accurately by placing it flat...than by clenching the mold." Wise [9] also noted that "increasing forces produced errors in the glove measurements, especially in the MCP joints." We observed through separate experimentation that in the flat hand position, the musculature in the hand tends to relax. In the grip position, in contrast, the muscles must maintain at least a minimal contraction in order to prevent dropping the mold. Varying levels of grip force can be applied, and the finger positions can be shifted slightly while still holding the mold closely. The measured variations in resistance easily accounted for the observed variations in the grip repeatability test results. The solution was to ask the individual to only grip the mold with enough strength to hold it, and that instruction will be given to future participants. While executing these tests can be challenging, we must concur that standardized testing is vital to ensuring that the collected data are useful and repeatable.

## Conclusion

The glove developed in this research is unconventional, and its uniqueness owes to the appropriate attention to the core requirements during the design analysis phase. The glove provides a novel method to evaluate actual functional capacity, starting with the dynamic evaluation of ROM as individuals participate in their normal daily activities.

The glove is not a generic solution, but a specific device to measure finger posture in an underserved population. Bend sensors were selected for their lightweight low profile, and for cost effectiveness. Although significant error can be introduced by using bend sensors, sensors with the appropriate repeatability characteristics have been identified. The bare Flexpoint sensors provided repeatable measurements with a 30 second error of 0.8% full scale, as compared to 9.5% for the next best solution, the Abrams-Gentile sensor. The overall glove configuration shows strong promise for providing repeatable measurements over long periods of time without undesired movement over the joints. Coefficient of variability averaged 2.96% and 0.10% across the five digits for the grip test and flat hand test, respectively. The glove is low cost; the total cost for the disposable portion of the device (glove material, adhesive and sensors) is less than $40, which is significantly less than any other reported solution. The glove can be used not only to measure flexion in individuals with reduced range of motion who cannot wear traditional measurement gloves, but also to measure passive ROM and cleaning activities in individuals who cannot initiate volitional activity in their affected hand.

Future directions include completion of the current study to establish repeatability and to identify calibration methods. The wireless link will be added midway through the study to provide full wearability and unencumbered movement, paving the way for extended studies in the home and community environments.

## Competing interests

The author(s) declare that they have no competing interests.

## Authors' contributions

LS made substantial contributions to the conception and design of the device and drafted the manuscript. DK identified a need for the device, contributed to the requirements and design of the device, and participated in revisions of the manuscript.

**Table 2:** Variability in glove measurements for repeatability Tests A and C

	**Test A: Grip Mold**			**Test C: Flat Hand**		
	
	Mean (Ohms)	SD (Ohms)	CoV	Mean (Ohms)	SD (Ohms)	CoV
Thumb	9138	50	0.55%	8257	15	0.18%
Index	14400	773	5.37%	8562	7	0.08%
Middle	27873	533	1.91%	7992	4	0.05%
Ring	51364	2368	4.61%	8231	6	0.07%
Pinkie	50629	1194	2.36%	7586	11	0.15%

## References

[B1] Finch E, Brooks D, Stratford PW, Mayo NE (2002). Physical Rehabilitation Ouctome Measures: A Guide to Enhanced Clinical Decision Making.

[B2] Dipietro L, Sabatini AM, Dario P (2003). Evaluation of an instrumented glove for hand-movement acquisition. Journal of Rehabilitation Research and Development.

[B3] Youngblut C, Johnson RE, Nash SH, Wienclaw RA, Will CA (1996). Review of virtual environment interface technology. (No IDA Paper P-3186): Institute for Defense Analyses, 1801 N Beauregard Street, Alexandria Virginia.

[B4] Karlsson N, Karlsson B, Wide P (1998). A glove equipped with finger flexion sensors as a command generator used in a fuzzy control system. Proceedings of the IEEE Instrumentation & Measurement Technology Conference 18–21 May 1998.

[B5] Zurbrügg T (2003). Dynamic Grasp Assessment for Smart Electrodes (GRASSY). Semester Thesis ETH Zurich (Swiss Federal Institute of Technology), Department of Information Technology and Electrical Engineering.

[B6] Hofmann F, Henz J (1995). The TU-Berlin Sensor Glove. Diploma thesis.

[B7] Jurgens J, Patterson PE (1997). Development and evaluation of an inexpensive sensor system for use in measuring relative finger positions. Med Eng Phys.

[B8] Simone LK, Elovic EP, Kalambur U, Kamper DG (2004). A low cost method to measure finger flexion in individuals with reduced hand and finger range of motion. Proceedings of the 26th Annual International Conference of the IEEE EMBS 1–5 September 2002.

[B9] Wise S, Gardner W, Sabelman E, Valainis E, Wong Y, Glass K, Drace J, Rosen JM (1990). Evaluation of a fiber optic glove for semi-automated goniometric measurements. Journal of Rehabilitation Research & Development.

[B10] Moerlein A Edward sensorhands. http://www.tufts.edu/programs/mma/emid/projectreportsS04/moerlein.html.

[B11] Morris SJ, Paradiso JA (2002). Shoe-integrated sensor system for wireless gait analysis and real-time feedback. Proceedings of the Second Joint EMBS/BMES 23–26 October 2002.

[B12] Cham JG, Stafford B, Cutkosky MR (2001). See labs run: A design-oriented laboratory for teaching dynamic systems. Proceedings of the 2001 ASME International Mechanical Engineering Congress and Exposition: 11–16 November 2001 New York.

